# Decoupling Conductivity, Heterogeneous Electron Transfer Rate, and Diffusion in Organic Molecular Electrocatalysis: Oxygen Reduction Reaction on Poly(3,4‐ethylenedioxythiophene)

**DOI:** 10.1002/smll.202409471

**Published:** 2024-12-15

**Authors:** Neha Sepat, Mikhail Vagin, Stefano Carli, Edoardo Marchini, Stefano Caramori, Qilun Zhang, Slawomir Braun, Zhixing Wu, Penghui Ding, Kosala Wijeratne, Ioannis Petsagkourakis, Ujwala Ail, Eleni Pavlopoulou, Tero‐Petri Ruoko, Simone Fabiano, Klas Tybrandt, Mats Fahlman, Reverant Crispin, Magnus Berggren, Viktor Gueskine, Isak Engquist

**Affiliations:** ^1^ Laboratory of Organic Electronics Department of Science and Technology Linköping University Norrköping 60174 Sweden; ^2^ Wallenberg Initiative Materials Science for Sustainability Department of Science and Technology Linköping University Norrköping 60174 Sweden; ^3^ Department of Environmental and Prevention Sciences‐DEPS University of Ferrara Ferrara 44121 Italy; ^4^ Department of Chemical Pharmaceutical and Agricultural Sciences‐DOCPAS University of Ferrara Ferrara 44121 Italy; ^5^ Bio and Organic Electronics Unit Department of Smart Hardware Digital Systems Division RISE Research Institutes of Sweden AB Norrköping 60221 Sweden; ^6^ Ligna Energy AB Bredgatan 33 Norrköping 60174 Sweden; ^7^ Institute of Electronic Structure and Laser Foundation for Research and Technology‐Hellas Heraklion Crete 71110 Greece; ^8^ Faculty of Engineering and Natural Sciences Tampere University Tampere 33720 Finland; ^9^ Wallenberg Wood Science Center Linköping University Norrköping 60174 Sweden

**Keywords:** 4‐ethylenedioxythiophene), nafion, oxygen reduction reaction, poly(3, poly(styrenesulfonate), secondary doping

## Abstract

The electrified production of hydrogen peroxide (H_2_O_2_) by oxygen reduction reaction (ORR) is attractive to increase the sustainability of chemical industry. Here the same chains of intrinsically conductive polymer, poly(3,4‐ethylenedioxythiophene) (PEDOT) are utilized, as ORR electrocatalyst, while varying polymeric primary dopants (PSS and Nafion) and the level of secondary doping with DMSO. These changes modulate various properties of the film, such as its microscale organization and electronic conductivity. The aim here is to clearly decouple the rate of the heterogeneous electron transfer (HET) of ORR from the diffusion affected by electronic conductivity and the electrochemically available surface area. It is found that the rate of HET and the double layer capacitance are significantly affected by primary dopant. On the contrary, secondary doping shows very little effect on the rate of HET. However, such secondary doping resulted in the increase of both electrochemically available surface area and the diffusion through the polymer film. This effect is attributed to a few orders increase of the electronic conductivity in the film improving availability of the polymer for electron transfer. The enhancement of diffusion upon the secondary doping of conducting polymer is utilized to improve direct conversion of air into H_2_O_2_ on gas diffusion electrode.

## Graphical abstract

The comparison of the electrode performance to drive oxygen reduction reaction on intrinsically conductive polymer PEDOT with different primary and secondary dopants enabled the decoupling of the rate of the heterogeneous electron transfer from the contribution of electrochemically available surface area. The diffusion enhancement is used to improve the gas diffusion electrode for direct conversion of oxygen from air to hydrogen electrode.

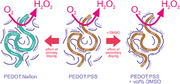

## 1 Introduction

H_2_O_2_ is a powerful and green liquid oxidant of industrial interest due to its high oxidative capability, as illustrated by the high equilibrium potential (*E_eq_* = +1.763 V (vs reversible hydrogen electrode (RHE) at pH 0) for the half‐reaction H_2_O_2_ + 2H^+^ + 2e^−^ → 2H_2_O, and water as its only product. Among a wide variety of applications,^[^ ^1^, ^2^, ^3^ ^]^ bleaching and recycling in the pulp and paper industry consumes 40% of the global production of H_2_O_2_, which exceeds 3 million tons per year. The traditional production of H_2_O_2_ by infrastructure‐dependent anthraquinone process relies on the use of organic solvents, hydrogen gas, and palladium‐based catalysts. Centralized production leads to high transportation costs of the concentrated peroxide to the utilization sites and security problems on the way.

Electrosynthesis of H_2_O_2_ via ORR using only abundant oxygen of air, water, and electricity offers a feasible de‐centralized alternative to the outdated production.^[^ ^4^, ^5^, ^6^ ^]^ The use of electricity from renewable sources, such as wind and solar power plants, for the production of chemicals, e.g. “green hydrogen peroxide”, contributes to UN Sustainable Development Goals^[^ ^7^ ^]^ and offers the possibility to reduce greenhouse gas emissions launching “green hydrogen peroxide”. Electrosynthesis of value‐added chemicals from Earth‐abundant feedstocks, such as air and water, is particularly attractive.

Electrosynthesis of H_2_O_2_ via two‐electron ORR (ORR‐to‐H_2_O_2_) can be written for basic media:

(1) $$O_{2}+2H_{2}O+2e^{-}⇄H_{2}O_{2}+2OH^{-}$$

(2)

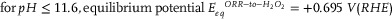



(3)

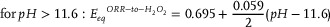




However, H_2_O_2_ is unstable. Firstly, it is an intermediate in the four‐electron ORR‐to‐water $\left(O_{2}+2H_{2}O+4e^{-}⇄4OH^{-}\right)$. The equilibrium potential of such reaction $\left(E_{eq}^{ORR-to-H_{2}O}=+1.23V\left(RHE\right)\right)$ is higher than for ORR‐to‐H_2_O_2_ at any pH implying the thermodynamic gain of complete four‐electron reduction in comparison with two‐electron ORR‐to‐H_2_O_2_. Secondly, fast disproportionation of H_2_O_2_ to oxygen and water catalyzed by transition metal ion contaminants is an additional reason of its instability.^[^ ^8^, ^9^ ^]^ Therefore, the electrosynthesis via ORR‐to‐H_2_O_2_ is feasible only under the conditions of kinetic control. The partial mitigation of the thermodynamic instability of H_2_O_2_ by de‐protonation at *pH* > 11.6^[^ ^10^ ^]^ allows to use inexpensive metal‐free electrode materials in basic media for electrosynthesis ORR‐to‐H_2_O_2_ for the needs of the pulp and paper industry.

Electrocatalysts allow to decrease electrical losses upon the direct transformation of electricity to chemical bonds and to control the process selectivity toward a certain intermediate or product. Electrocatalysts for ORR‐to‐H_2_O_2_ are based on scarce platinum group metals and mercury‐based compounds, which could possibly release toxic species.^[^ ^11^, ^12^ ^]^ The reported efficient carbon‐based ORR‐to‐H_2_O_2_ electrocatalysts^[^ ^13^, ^14^, ^15^, ^16^, ^17^ ^]^ have uncertain active sites, which complicates the elucidation of their structure–performance relationship and performance optimization.^[^ ^18^ ^]^ True ORR electrocatalysis leading to the reduction of activation barriers in the multistep ORR is assured by chemisorption of oxygen, a triplet di‐radical molecule, which relies on the presence of the unsaturated valences at the surface of catalytic atomic crystals.^[^ ^19^, ^20^, ^21^, ^22^ ^]^

Alternatively, organic molecular systems, such as conducting polymers,^[^ ^23^ ^]^ are not prone to chemisorb a radical species like oxygen molecule and proceed via outer‐sphere electron transfer.^[^ ^23^ ^]^ This favors the selectivity to ORR‐to‐H_2_O_2_.

Conducting polymers, which are prepared by low‐temperature synthesis from abundant elements, constitute another class of electrode materials. Electronic conductivity in a *p*‐type conducting polymer is assured by positive carriers compensated by anions, the so‐called primary dopants. Therefore, in an electrolyte solution a conducting polymer film behaves as mixed ion‐electron conductor. Specifically, when either the electrical charge within the film is modulated by external electronics or the ionic charge by ion concentration gradient, this leads to compensational ionic or electronic transport in the bulk of the film, respectively, to maintain the electroneutrality of the film. Porosity of conducting polymers at the molecular scale facilitates the access of both ions forming an electrical double layer and neutral reagents to individual backbones,^[^ ^24^, ^25^ ^]^ which results in the capacitance and HET distributed throughout the film bulk. It is the electron transfer that is relevant for ORR‐to‐H_2_O_2_.^[^ ^23^, ^26^, ^27^, ^28^, ^29^, ^30^ ^]^

**Scheme 1:**
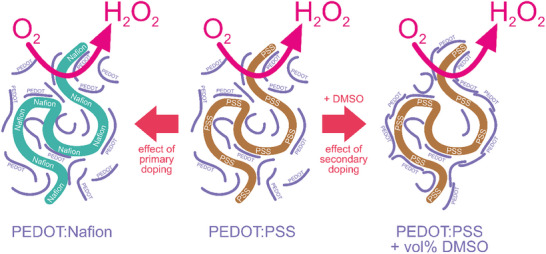
The primary and secondary doping of PEDOT films on ORR.

Poly(3,4‐ethylenedioxythiophene) (PEDOT) is an archetypical conducting polymer with pH‐independent electronic conductivity. PEDOT composites with poly(styrenesulfonate) as the primary dopant (PEDOT:PSS) in aqueous colloidal form are commercially available and suitable for the fabrication and even printing of stable films with high electronic and ionic conductivities.^[^ ^31^, ^32^ ^]^ Primary dopants are the counter ions compensating the free carriers of electrical charge of the conducting polymer phase. Specifically, the positive charge carriers in p‐type conducting polymers such as PEDOT, are compensated by anionic primary dopants. Both immobile poly‐anions (**Scheme** **1**) and small exchangeable anions can be primary dopants of PEDOT. Here, in parallel to the standard PEDOT:PSS, we study PEDOT prepared with a different poly‐sulfonate dopant, namely Nafion.

At the meso‐scale, electronic transport in conducting polymers is strongly dependent on microscale morphology and self‐assembly of polymer chains, as it is maintained via hopping between conjugation‐rich conductive islands isolated in a less conducive matrix.^[^ ^32^ ^]^ Treatment that increases the electrical conductivity of a conducting polymer without changing its oxidation level is called secondary doping (Scheme 1). The increase of the hopping rate is arguably due to the formation of larger conducting islands, reduced size of the insulating regions that hinder electron transfer^[^ ^33^, ^34^ ^]^ and leads to percolation path elongation and conductivity increase.^[^ ^35^, ^36^ ^]^ It is the addition of a high‐boiling solvent as the secondary dopant into the PEDOT:PSS blend that increases the phase separation in the resulting film, which nevertheless contains no more secondary dopant after drying. Among the variety of secondary dopants,^[^ ^37^, ^38^, ^39^, ^40^, ^41^, ^42^, ^43^, ^44^ ^]^ we selected DMSO in this work.

For the same conducting polymer molecule, the change of the primary dopant and the level of secondary doping can influence various properties of the resultant material, not only its electronic conductivity, which traditionally attracted particular attention. An electrochemical reaction at a conducting polymer is a complex process involving charge transfer at and mass transport (diffusion) to, from, and inside this porous electrode. It is thus not sufficient to register that one material performs better than the other but to understand precisely which property caused the result. In this work, we aim the clear demarcation of the effects on HET kinetics from the effects on diffusion modulated by electrochemically available surface area (EASA).

We are able to show that the rate of the HET is almost independent of secondary doping for PEDOT:PSS, in spite of few orders increase of its electronic conductivity. However, the change of primary dopant of PEDOT from PSS to Nafion is shown to result in notable increase of both HET rate and the double layer capacitance. But the consequence of the secondary doping is the increase of EASA and apparent diffusion coefficient through the electrocatalyst film. This was used to improve the performance of a gas diffusion electrode enabling the direct conversion of oxygen of air.

We believe that the conservation of surface state on conducting polymer in parallel with the change of primary and secondary doping enables the general model of molecular electrocatalysts.

## 2 Results and Discussion

### 2.1 Structure of PEDOT:PSS and PEDOT:Nafion

**Figure 1:**
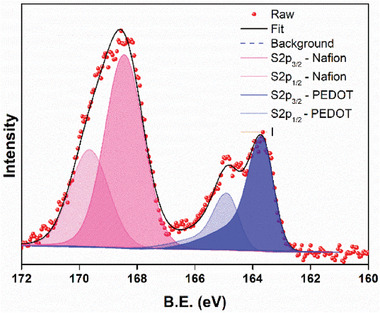
The XPS core level signal of sulfur in PEDOT:Nafion.

The primary doping induced features of PEDOT:Nafion are revealed by XPS and Raman spectra. The XPS core level signal of sulfur showed two contributions from a doublet from PEDOT at low binding energy (167–163 eV) and the other contribution at higher binding energy (171–167 eV) that can be ascribed to sulfonate groups of Nafion dopant (**Figure** **1**). The presence of positive charges delocalized over doped PEDOT chains results in a shift of the core levels to higher binding energy with respect to neutral PEDOT, which is visualized by the strong asymmetry of the S2p core level signal. The obtained spectra are similar to those reported for PEDOT:PSS.^[^ ^45^ ^]^ Fitting reveals that the doping ratio of PEDOT is 0.3, which is also a usual value for PEDOT:PSS. Analysis of the ratio between Nafion S2p peaks and that of PEDOT gives the ratio between Nafion and EDOT monomer units as 1.6, indicating an excess of Nafion, which is significantly smaller than the 3‐fold excess reported for PEDOT:PSS.

From this data we can calculate the content of the conducting polymer PEDOT in both PEDOT:PSS and PEDOT:Nafion compositions we will need for the catalyst activity characterization. The molecular masses of SS and EDOT monomer units are 183 and 140 g mol^−1^, the estimated equivalent mass of Nafion is 1100 g mol^−1^.^[^ ^46^ ^]^ The mass shares of PEDOT calculated from the molar ratios EDOT:counterion of 1:3 in PEDOT:PSS and 1:1.6 in PEDOT:Nafion are thus translated into 140/(140+3×189) = 19.8% mass for PEDOT:PSS and 140/(140 + 1.6 × 1100) = 7.4% mass for PEDOT:Nafion.

Raman analysis was performed to confirm the highly doped state of PEDOT:Nafion. In particular, the Raman spectra of PEDOT:Nafion film (Figure S1B, Supporting Information) is dominated by the strong signal at 1411 cm^−1^, which refers to the *C_α_* = *C_β_* symmetric stretching vibrations of the neutral and oxidized form structures, respectively.^[^ ^47^, ^48^ ^]^ This signal is shifted by ∼11 cm^−1^ to lower wavenumber (1400 cm^−1^) upon chemical reduction of the PEDOT:Nafion film. It was reported that a blue shift of the *C_α_* = *C_β_* symmetric stretching vibration peak with respect to the reduced and undoped form of PEDOT:PSS is consistent with the formation of highly doped conductive polymer.^[^ ^47^, ^49^ ^]^ Thus, this confirms that PEDOT:Nafion is present in its highly doped state, in accordance with XPS analysis. In addition, the typical signature for polarons and bipolarons as charge carriers can be displayed in the absorption spectra of PEDOT:Nafion dispersion (Figure S1A, Supporting Information). This spectrum is characterized by the absence of any absorption peak between 400 and 800 nm and by a progressive rise of the absorbance at higher wavelength. This further corroborates the highly doped state of PEDOT:Nafion.^[^ ^46^ ^]^

The structural origin of conductivity's enhancement in PEDOT:PSS films upon DMSO addition is well documented in literature.^[^ ^35^ ^]^ The GIWAXS scattering patterns acquired for various amounts of the secondary dopant DMSO (Note S1 and Figure S3, Supporting Information) showed the presence of two major peaks at 1.32 and 1.82 Å^−1^, which are assigned to the amorphous halo of PSS and to the π‐π stacking of PEDOT,^[^ ^32^, ^35^, ^50^ ^]^ respectively. As DMSO quantity increases, the scattered intensity increases equally for the two peaks. Following literature, this can be interpreted as a larger phase separation between PEDOT domains and PSS matrix and increase in the degree of order of the PEDOT crystals, which is favorable for efficient charge conduction.^[^ ^35^, ^51^ ^]^

### 2.2 Secondary Doping Effect on the Conductivity of PEDOT:PSS

**Figure 2:**
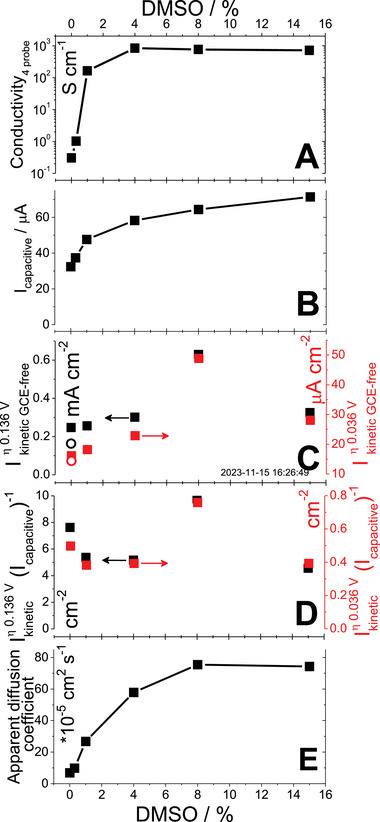
The effect of secondary doping (DMSO content in PEDOT:PSS blends) on four‐probe conductivity (A), on EASA (capacitive currents, B), on ORR kinetic currents (C) at low overpotentials (open symbols – PEDOT:Nafion, filled symbols – PEDOT:PSS; η 0.036 V (red symbols, applied potential 0.7 V (RHE)) and η 0.136 V (black symbols, applied potential 0.6 V (RHE)), on ORR kinetic currents normalized by capacitive currents (D) at low overpotentials (η 0.036 V and η 0.136 V as red and black symbols, respectively) and the apparent diffusion coefficient of oxygen (E).

To reveal the secondary doping effect, we measure the electrical conductivity of PEDOT:PSS films. In dry conditions, we utilized ex situ measurements by four‐point‐probe technique (**Figure** **2**A). The increase of secondary dopant content in the blend used for the film preparation led to more than three orders increase of electrical conductivity of PEDOT:PSS film (from 0.3 S cm^−1^ for 0 vol% to 838 S cm^−1^ for 4 vol% of DMSO in the blend), which is consistent with our previous findings.^[^ ^52^ ^]^ As the addition of DMSO does not enhance electron transfer, the increase of conductivity can only be due to morphological changes, namely the enhanced phase separation between PEDOT and PSS domains.

**Figure 3:**
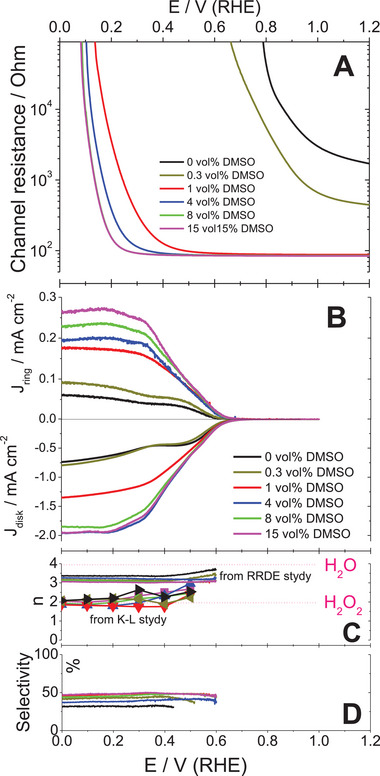
The potential dependencies of in situ resistance of the PEDOT:PSS films (A), ring and disk currents (B) recorded on a platinum ring electrode and on a glassy carbon disk electrode modified by PEDOT:PSS blends with different DMSO content (oxygen‐saturated 0.1 m KOH (pH 13), 1600 rpm, scan rate 20 mV s^−1^), estimated number of electrons transferred on oxygen molecule (C) and ORR selectivity (D).

To evaluate the effect of secondary doping on the conductivity of the PEDOT at the conditions of ORR electrocatalysts, we performed in situ resistometry (**Figure** **3**A) on the electrochemical transistor device with PEDOT:PSS films as channel material immersed into aqueous electrolyte (0.1 m KOH). The modulation of potential applied between conducting polymer film and reference electrode led to significant changes in the channel resistance. The high degree of film oxidation, that is a high doping level of PEDOT, is reached at the applied potentials of ca. 1–1.2 V, as evidenced by decreased channel resistance. At applied potentials below 0.8 V a few orders of magnitude increase in channel resistance indicates film reduction, so‐called de‐doping. Importantly, the effect of secondary doping is also clear. As DMSO content in the PEDOT:PSS blend increases, the potential window of high conductivity broadens, in coherence with cyclic voltammetry data acquired in neutral aqueous electrolyte (Figure S4, Supporting Information). The films formed from the blends of the lowest DMSO contents (0 vol% and 0.3 vol% of DMSO) showed significantly higher film resistances even at high positive potentials. The coherent behavior has been observed on organic electrochemical transistor operated in neutral electrolyte (Note S2, Supporting Information).

The effect of secondary doping with DMSO is truly morphological and decoupled from the increase in primary doping level, i.e., free from alteration of electrical charge on the conjugated backbone. Then the observed extension of the high conductivity window is rather due to the increase in the density of percolation paths for electronic transport within the bulk of the film. At the same time, this implies that a higher fraction of the film takes part in the establishment of the electrical double layer, which is formed at the physical boundary between the electronic and ionic conductors. In other words, the secondary doping extends the surface of the electrode at which electrical double layer is formed, namely EASA, within the porous bulk of the PEDOT:PSS film.

### 2.3 Capacitance and Electrochemically Active Surface Area (EASA) of PEDOT:PSS and PEDOT:Nafion

Capacitive currents are the manifestation of capacitive EASA. The capacitive currents recorded on PEDOT:PSS‐modified glassy carbon electrodes by voltammetry in electrolyte‐filled three‐electrode cell grow with the increase of DMSO content in the blend (Figure 2B), in coherence with in situ resistometry reported above. Notably, the EASA of PEDOT:PSS films scales up linearly with increase of the film thickness. The increase of PEDOT:PSS blend loading on the glassy carbon resulted in a linear increase of capacitive currents (Figure S7, Supporting Information). Coherently, the impedance measurements of PEDOT films in the capacitive region (Note S3 and Figures S8–S10, Supporting Information) showed a linear scaling‐up of the film capacitance with the mass loading of the films (Figure S8B, Supporting Information) and a similar effect of the secondary doping. This implies that the bulk of the film is accessible for the electrocapacitive process due to its porosity at the molecular scale.

The effect of the primary dopant manifests as more than 3‐fold increase of total film capacitance obtained for PEDOT:Nafion film in comparison with PEDOT:PSS film of similar mass fabricated from the DMSO‐free blend (Figure S8C, Supporting Information). In accordance with XPS data, the concentration of electronic conductor, namely PEDOT, is almost two times smaller in PEDOT:PSS than in PEDOT:Nafion. Therefore, 2‐times diluted conductor (PEDOT:PSS) showed 3‐times lower total capacitance. Such incoherency manifests the higher density of surface states created by Nafion doping with respect to PSS doping on PEDOT‐based films of equal masses.

On the contrary, the secondary doping of PEDOT:PSS leads to values of total capacitance similar to those obtained for PEDOT:Nafion.

### 2.4 ORR on PEDOT:PSS and PEDOT:Nafion Electrodes

#### 2.4.1 Number of Electrons Transferred Per Oxygen Molecule

Having characterized our electrode films, we are able to discuss the ORR results obtained on them. We focused on measurements in an alkaline electrolyte. This is because of our preliminary experiments showed a surprisingly strong pH dependence of ORR activity on PEDOT: no ORR‐associated currents were detected on PEDOT:PSS‐modified electrode in aqueous acidic electrolyte (Figure S12, Supporting Information). Comparing ORR on PEDOT:PSS coated and blank glassy carbon disk electrodes, it is important to note that ORR‐associated disk and ring currents at both are quite close (Figure S13, Supporting Information). In order to de‐couple the PEDOT:PSS activity from that of the glassy carbon substrate, it was essential to study the dependence of kinetic currents on PEDOT:PSS film thickness.

The *mass‐normalized* ORR kinetic currents *decrease* as PEDOT:PSS coating gets thicker, for all secondary doping levels (Figure S14, Supporting Information). At the same time, their *capacitive EASA* measured in the background electrolyte *scales up linearly* with the film thickness. We conclude that the PEDOT:PSS film bulk is less accessible for ORR than its surface, that is the *ORR EASA* of such films is different from their *capacitive EASA*. Indeed, capacitive EASA reflects the total electrical double layer formed by ions attracted from solution into the bulk of porous coating to compensate the charges in the film. But the penetration of neutral oxygen inside the coating is not coulombically driven, which curbs ORR EASA compared to capacitive EASA. Furthermore, the low values of ORR‐associated mass‐normalized kinetic current on thicker PEDOT:PSS films suggest minimized contribution from the underlying glassy carbon, while for thin films the ORR activity of the substrate could not be neglected. Consequently, we decided to utilize the kinetic currents obtained for thick PEDOT:PSS films in our further analysis.

We utilized hydrodynamic voltammetry on rotating ring‐disk electrode (RRDE) to distinguish the currents limited by diffusion from those limited by HET kinetics. Moreover, such approach allows the estimation of the average number electrons consumed in reduction per oxygen molecule. The glassy carbon disc was modified either with PEDOT:PSS or PEDOT:Nafion. An independent platinum ring electrode fencing the disc was positively polarized at 1.2 V (RHE) to drive H_2_O_2_ oxidation and served as detector quantifying the in‐situ yield of H_2_O_2_.

In oxygen‐saturated electrolyte, (A) typical S‐shaped waves of negative currents were recorded on the disk electrode (Figure 3B) and (B) positive currents (H_2_O_2_ oxidation) were recorded on the ring. These observations together testify the proceeding of ORR‐to‐H_2_O_2_, namely reaction (1).

The diffusion‐independent ORR kinetic currents, which originated from HET, were obtained by Koutecky–Levich (KL) analysis of the entire family of background‐corrected (vs oxygen‐free electrolyte) voltammograms recorded on the rotating disk at different rotations speeds (Figure S15 and Note S4, Supporting Information). The KL number of transferred electrons per oxygen molecule is always found close to 2 (Figure 3C) indicating that the two‐electron pathway (ORR‐to‐H_2_O_2_) dominates regardless the secondary doping level of PEDOT:PSS in alkaline media. This mechanistic observation is coherent with the results from our previous studies on different PEDOT films.^[^ ^27^, ^53^ ^]^ However, independent quantification of the ring currents (Note S5, Supporting Information) yields the number of transferred electrons per oxygen molecule close to 3 (Figure 3C), which deviates from 2 expected for ORR‐to‐H_2_O_2_. This points to the selectivity of ORR, namely in situ H_2_O_2_ yield, significantly lower than 100% (Figure 3D). This discrepancy could be due to the fast disproportionation of H_2_O_2_ on the traces of transition metal ions always present in the bulk of aqueous alkaline electrolyte prepared from solid hydroxides.^[^ ^8^, ^9^ ^]^

### 2.5 Tafel Slope and Mechanism

**Figure 4:**
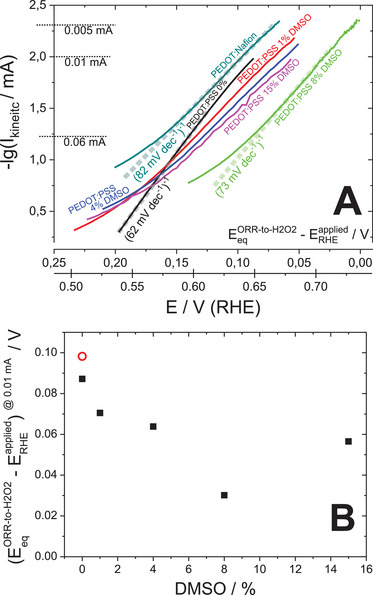
The effect of primary and secondary doping on kinetics of ORR‐to‐H_2_O_2_ on PEDOT. A: the dependencies of the logarithm of ORR kinetic current on the overpotential (Tafel plots) obtained on PEDOT:Nafion and PEDOT:PSS films fabricated from blends with different DMSO content; B: the dependence of ORR overpotential at 0.01 mA obtained for PEDOT:Nafion and PEDOT:PSS films (open red symbols – PEDOT:Nafion, filled black symbols – PEDOT:PSS).

The overpotential (η) for a process is the difference between the equilibrium potential and the applied potential ($\eta =E_{eq}^{ORR-to-H_{2}O_{2}}\;-E_{RHE}^{applied}$; for $E_{RHE}^{applied}=0.7$ V (RHE): η  =  0.036 V), namely the driving force for the reaction. We plotted the kinetic currents (from KL analysis) obtained for various secondary doping levels of PEDOT:PSS in Tafel coordinates (**Figure** **4**A), i.e., the dependence of logarithm of kinetic current *lg*(*I_kinetic_*) on overpotential. The clear linearity in Tafel coordinates implies that the kinetic current, that is the rate of the HET, increases exponentially with the driving force at the small overpotentials, which confirms the pure kinetic control. High overpotentials (ca. η > 0.2 *V*) are less reliable for the kinetic analysis due to possible limitation by diffusion in the pores of PEDOT:PSS.

All the PEDOT:PSS films except “pristine” (0 vol% DMSO) PEDOT:PSS showed the same reciprocal Tafel slope (Figure 4A: all the curves are parallel at low overpotentials) implying that the ORR mechanism remains the same regardless of the secondary doping level. The values of Tafel slope estimated for all PEDOT‐based systems were in the range of 62 mV dec^−1^ (pristine PEDOT:PSS 0% DMSO) and 82 mV dec^−1^ (PEDOT:Nafion). These are close to 60 mV dec^−1^ assigned to the $E\bar{C}E$ mechanism,^[^ ^54^ ^]^ where the first and the second electron transfers of ORR‐to‐H_2_O_2_ are separated by the slow chemical (free from electron transfer) reaction ($\bar{C})$, which is the rate‐determining step. It is computed that such slow chemical reaction can be protonation of the product of the first electron transfer ($O_{2}^{-}+H_{2}O⇄O_{2}H+OH^{-}$).^[^ ^55^ ^]^ The slightly higher value of the Tafel slope observed for ORR on PEDOT:Nafion might imply the alteration of the mechanism.

All these results indicate that electrical conductivity of PEDOT:PSS; which is significantly modified by secondary doping, has no effect on the ORR mechanism on it.

### 2.6 Normalization of ORR Kinetic Currents

With a different primary dopant, Nafion instead of PSS‐anion, and in the absence of secondary doping, only a minor increase in the ORR threshold overpotential to attain the kinetic current of 0.01 mA was observed: from 0.087  to 0.098 V (Figure 4B). ORR kinetic currents at low overpotentials were also slightly lower with Nafion as the primary dopant compared to PSS in the absence of secondary doping (Figure 2C). But the secondary doping of PEDOT:PSS resulted in a notable decrease of this overpotential, from 0.087 V down to 0.03 V (Figure 4B), which could be due to lower voltage losses in the more conductive films. The minimum value of overpotential observed for PEDOT:PSS film fabricated from the blend with 8 vol% DMSO (Figure 4B) could correspond to the optimum between capacitive EASA and ORR EASA of PEDOT:PSS available for the HET. To clarify this, we investigated separately the effects of EASA and PEDOT:PSS mass on ORR kinetic currents.

The kinetic (diffusion‐free) currents at low ORR‐to‐H_2_O_2_ overpotentials (0.136 *V* ≥ η ≥ 0.036 *V*) on thick PEDOT:PSS films of identical masses increased insignificantly with their secondary doping level(Figure 2C). However, when normalized by their EASA‐associated capacitive currents (Figure 2B), ORR kinetic currents showed no pronounced effect of secondary doping level (Figure 2D), in spite of many orders increase of their conductivity (Figure 2A). Similar results were obtained by electrochemical impedance spectroscopy on stagnant PEDOT films (Note S3 and Figure S11, Supporting Information). To reiterate, the conductivity of the PEDOT:PSS film has no effect on the rate of the HET, provided that the EASA at which this electron transfer takes place is considered. The secondary doping creates no additional active sites to enhance ORR kinetics, but only increases the EASA. This result manifests the decoupling of bulk and surface properties of PEDOT:PSS films, electronic conductivity and the surface states, respectively.

**Figure 5:**
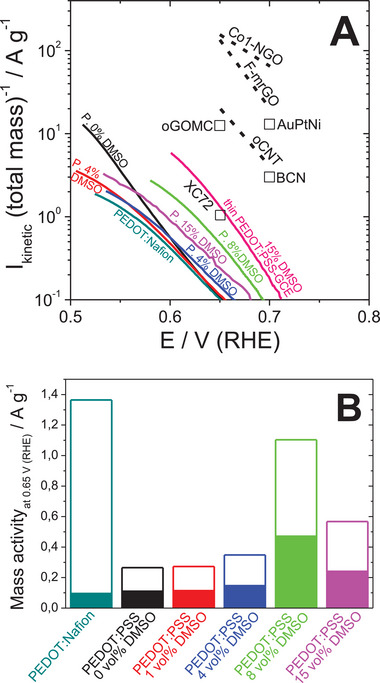
The effect of primary and secondary doping of PEDOT on the ORR‐to‐H_2_O_2_ mass activity. A: comparison of the potential dependencies of kinetic currents normalized on the mass of the catalysts at different potentials obtained on different PEDOT films and on the reported ORR‐to‐H_2_O_2_ catalysts in 0.1 M KOH (Co1‐NGO (cobalt‐incorporated nitrogen‐doped graphene);^[^ ^13^ ^]^ F‐mrGO (few‐layered mild‐reduced graphene oxide),^[^ ^14^ ^]^ AuPtNi (Au‐Pt‐Ni nanorods), oCNT (oxidized carbon nanotubes), BCN (boron carbon nitride), oGOMC (graphitic ordered mesoporous carbon), XC72 (Vulcan XC72), thin PEDOT:PSS on GCE); B: effect of primary and secondary doping of PEDOT on ORR‐to‐H_2_O_2_ mass activity (kinetic currents normalized by total mass of catalyst and by mass of electronic conductor – filled and open columns, respectively).

The mass activities, that is, the ORR kinetic currents normalized by the *total* film mass, of all thick PEDOT films were lower than the performance of reported state‐of‐the‐art ORR‐to‐H_2_O_2_ electrocatalysts evaluated in identical conditions (**Figure** **5**A). For PEDOT films, these were close to the performance of commercial beaded carbon black, Vulcan XC72.^[^ ^14^ ^]^ The mass activity we reported previously for thin PEDOT:PSS film^[^ ^53^ ^]^ could be overestimated due to the contribution from the underlying glassy carbon. Comparing the mass activity of PEDOT:Nafion and PEDOT:PSS in the absence of secondary doping, we observe no effect of the primary dopant (Figure 5A). However, as the electrons can only be transferred via the conducting polymer, and not the counterion, it is instructive to normalize the activity by the mass of PEDOT only, excluding PSS or Nafion, based on our XPS data. As the mass share of PEDOT in PEDOT:PSS or PEDOT:Nafion is 19.8% and 7.4% mass, respectively, the increase in the mass activity per PEDOT with respect to total would be 5 and 13.5 times. The results are shown in Figure 5B. Here the effect of the primary dopant is visible. The ORR‐to‐H_2_O_2_ kinetic current at a low driving force (at small overpotential of 0.65 V (RHE); η  =  0.086 V)) normalized on the mass of the actual conducting polymer in the composite shows more than 5 times increase upon the change of primary dopant from PSS to Nafion.

Considering the mass of the electronic conductor in the catalyst films we can conclude that the change of primary dopant of PEDOT from PSS to Nafion coherently increases both the mass‐normalized kinetic current of ORR and the mass‐normalized double layer capacitance. The coherent increase of both the HET rate and the electrode capacitance due to the increase of the density of surface states is reported for carbon materials.^[^ ^56^ ^]^ Therefore, the effect of primary doping can be explained by the increase of the density of surface states.

In parallel, optimization of capacitive EASA by the secondary doping of PEDOT:PSS enables to achieve a 30% higher mass activity than on PEDOT:Nafion.

At high overpotentials (η ≥ 0.5 *V*), the ORR currents are limited by the diffusion of reagents and products. These currents were significantly affected by the secondary doping level of PEDOT:PSS films (Figure 3B), in contrast to the kinetic currents measured at the low overpotentials: the increase in the DMSO content lead to more than 2‐fold increase in the disk currents recorded at potentials below 0.2 V (RHE). At the same time, the increase in H_2_O_2_ production was more than 5‐fold, according to the currents recorded on the platinum ring detector. It is instructive to perform Levich analysis (Note S6 and Figure S16, Supporting Information) to the limiting currents at different secondary doping levels of PEDOT:PSS. The diffusion coefficient of the reactant can be obtained from the Levich equation (5S) only based on certain assumptions. First, we assume the number of transferred electrons equal to 2 (for ORR‐to‐H_2_O_2_). Secondly, we assume the geometrical area of the disk as the electrode surface. The diffusion coefficients obtained in this way at the potential of 0.1 V (RHE) on PEDOT:PSS films increase by more than one order of magnitude with secondary doping level (Figure 2E) up to the values by far exceeding the diffusion coefficient of oxygen molecule in water (2.4 × 10^−5^ cm^2^ s^−1^), which normally cannot be increased by electrode modification. Aware of this, we call it “apparent diffusion coefficient” and attribute its growth with secondary doping to the increase of actual EASA. This value highlights an important origin of higher currents obtained at modified electrodes irrespective of their electrocatalytic properties. EASA largely exceeds the geometric area of the electrode due to the presence of the porous polymer film.

### 2.7 Gas Diffusion Electrode (GDE)

**Figure 6:**
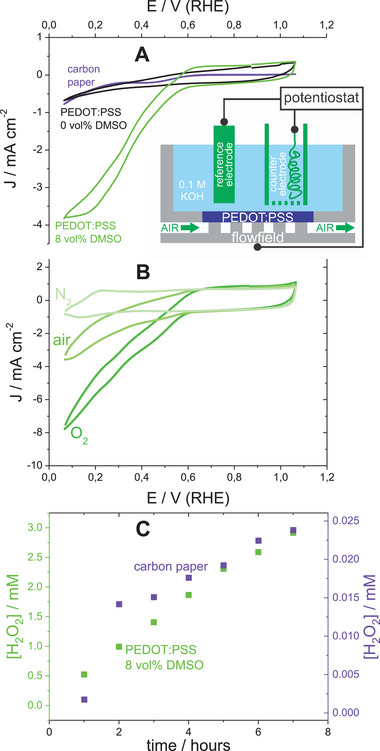
The direct electrosynthesis of H_2_O_2_ from air on PEDOT:PSS‐based GDE. A: cyclic voltammograms recorded with an air‐fed GDE assembled with pristine carbon paper and with carbon paper modified by PEDOT:PSS without and with secondary doping (0 vol%. and 8 vol% DMSO, respectively; 0.1 m KOH); **Inset**: scheme of the GDE cell; B: cyclic voltammograms recorded on nitrogen‐, air‐ and oxygen‐fed GDEs assembled with PEDOT:PSS‐modified carbon paper (8 vol% DMSO, 0.1 m KOH); C: the time dependencies of electrosynthesized H_2_O_2_ concentration obtained on pristine and PEDOT:PSS‐modified carbon papers.

The cell design equipped with a GDE (Inset in **Figure** **6**A) overcomes the limitation by low oxygen solubility in the aqueous electrolytes (ca. 1 mm) by direct use of air containing ca. 21 mol% of oxygen. We modified porous carbon paper GDE with secondary doped PEDOT:PSS films in order to utilized the enhanced diffusion through this conducting polymer material for direct diffusion‐controlled electrosynthesis of H_2_O_2_ from air as source of oxygen. The major effect of secondary doping is indeed visible as an increase in ORR‐associated currents in the region of diffusion control (e.g., 0.2 V (RHE), Figure 6A) on an air‐fed GDE with PEDOT:PSS, if we compare the blend with 8% DMSO and pristine PEDOT:PSS (0% DMSO). Moreover, the pristine PEDOT:PSS led to the full suppression of ORR currents observable at the blank bare carbon paper GDE (Figure S17, Supporting Information), in spite of the capacitive EASA increase due to PEDOT:PSS. Therefore, pristine PEDOT:PSS acts as an inert ORR‐inactive electronic conductor. It is the secondary doping that results in the appearance of ORR‐associated currents at the potentials of diffusion control. The change of the gas fed from air to oxygen led to the archetypical increase in diffusional ORR currents (Figure 6B), which clearly demonstrates that the limitation is due to oxygen diffusion, not reaction kinetics. These observations allow us to conclude that the secondary doping of PEDOT:PSS switched the slowest diffusion process from intrinsic charge transport maintained by both electronic and ionic diffusion to oxygen diffusion. The dependence of current (e.g., at 0.15 V (RHE)) on the oxygen content (namely, 0%, 21%, and 100% for pure nitrogen, air, and pure oxygen, respectively) is five times steeper on secondary doped PEDOT:PSS in comparison with blank carbon paper (data not shown). This once again demonstrates that oxygen diffusion is enhanced on PEDOT:PSS carbon paper compared to blank (non‐modified) carbon paper.

We further quantified the electrosynthesis of H_2_O_2_ on PEDOT:PSS‐modified GDE by constant current electrolysis. The modification of carbon paper by secondary doped PEDOT:PSS led to a significant improvement in the H_2_O_2_ production rate (Figure 6C). After 7 h of electrolysis at 5.7 mA cm^−2^, more than two orders of magnitude more peroxide were obtained on the PEDOT:PSS modified than on blank carbon‐paper GDE. Coherently, the modification of carbon paper with this conducting polymer blend resulted in a decrease in voltage (Figure S18, Supporting Information), which we attribute to the mitigation of voltage loss due to limited diffusion on blank carbon paper. The Faradaic efficiency of the H_2_O_2_ electrosynthesis was still below 50% and almost independent of the current density (Figure S19, Supporting Information), and it slightly decreased with elapsed time of operation (Figure S20, Supporting Information). The low efficiency of electrosynthesis could be due to the subsequent reduction of H_2_O_2_.

The minimum electrical energy consumption we attained was ca. 8 kWh per kg of H_2_O_2_. To put in perspective, one can estimate the Gibbs free energy of the reaction considering the overall process in a GDE cell as the comproportionation reaction $H_{2}O_{l}+\frac{1}{2}O_{2}\to H_{2}{O_{2}}_{l},\Delta \;G_{reaction}=\Delta G_{H_{2}{O_{2}}_{l}}\;-\Delta \;G_{H_{2}O_{l}}=-120\;kJ\;mol^{-1}-\;\left(-237\;kJ\;mol^{-1}\right)$ = 117 *kJ mol* ^−1^ or 0.96 kWh per kg of H_2_O_2,_ which corresponds to the lowest, thermodynamic limit. Therefore, the operation of the PEDOT‐based GDE in direct H_2_O_2_ electrosynthesis from the air has an 8‐fold loss in energy from the thermodynamic limit (Figure S20, Supporting Information). The losses likely originate from parallel unproductive Faradaic processes and complex diffusion on GDE.

## 3 Conclusion

We compared the performance of PEDOT electrodes with PSS and Nafion polymeric counter‐ions as primary dopants and PEDOT:PSS at different secondary dopant levels in ORR‐to‐H_2_O_2_ electrocatalysis. We were able to decouple the HET rate in ORR from the contribution of electrochemically available surface area (EASA) to the overall ORR current. The change of the primary dopant from PSS to Nafion increases both HET of ORR and double layer capacitance, which can be articulated as the increase of the density of the surface states. On the contrary, the effect of secondary doping was limited to the increase in the EASA and apparent diffusion throughout the electrocatalytic interface due to the increase in conductivity of the conducting polymer, while its effect on the HET rate was minor. However, the EASA available for ORR lags behind the capacitive EASA at which an electric double layer is formed. The diffusion enhancement at the interface due to modification with PEDOT:PSS was utilized to improve the gas diffusion electrode for direct conversion of oxygen from air to H_2_O_2_.

## Conflict of Interest

The authors declare no conflict of interest.

## Supporting information



Supporting Information

## Data Availability

The data that support the findings of this study are available from the corresponding author upon reasonable request.
