# Supplementary material for: Decoupling Conductivity, Heterogeneous Electron Transfer Rate, and Diffusion in Organic Molecular Electrocatalysis: Oxygen Reduction Reaction on Poly(3,4‐ethylenedioxythiophene)
Source: Small. 2024 Dec 15;21(5):2409471. doi: 10.1002/smll.202409471 (PMC11798352; doi:10.1002/smll.202409471)
Supplement: Supplementary file 1 — Supporting Information [file SMLL-21-2409471-s001.docx]

**Supporting Information**

**Decoupling conductivity, heterogeneous electron transfer rate, and diffusion in organic molecular electrocatalysis: oxygen reduction reaction on poly(3,4-ethylenedioxythiophene)**

Neha Sepat^1^, Mikhail Vagin^1,2^*, Stefano Carli^3^, Edoardo Marchini,^4^ Stefano Caramori,^4^ Qilun Zhang^1^, Slawomir Braun^1^, Zhixing Wu^1^, Penghui Ding^1^, Kosala Wijeratne^1^, Ioannis Petsagkourakis^1,6^, Ujwala Ail^7^, Eleni Pavlopoulou^8^, Tero-Petri Ruoko^9^, Simone Fabiano^1,2^, Klas Tybrandt^1,2,5^, Mats Fahlman^1^, Reverant Crispin^1,2,5^, Magnus Berggren^1,2,5^, Viktor Gueskine^1,5^, Isak Engquist^1,5^

^1^Laboratory of Organic Electronics, Department of Science and Technology, Linköping University, 60174 Norrköping, Sweden

^2^Wallenberg Initiative Materials Science for Sustainability, Department of Science and Technology, Linköping University, Norrköping 60174, Sweden

^3^Department of Environmental and Prevention Sciences-DEPS, University of Ferrara, 44121 Ferrara, Italy

^4^Department of Chemical, Pharmaceutical and Agricultural Sciences*-*DOCPAS, University of Ferrara, 44121 Ferrara, Italy

^5^Wallenberg Wood Science Center, Linköping University, 60174 Norrköping, Sweden

^6^Bio and Organic Electronics Unit, Department of Smart Hardware, Digital Systems Division, RISE Research Institutes of Sweden AB, 60221, Norrköping, Sweden

^7^Ligna Energy AB, Bredgatan 33, 60174, Norrköping, Sweden

^8^Institute of Electronic Structure and Laser, Foundation for Research and Technology-Hellas, 71110 Heraklion, Crete, Greece

^9^Faculty of Engineering and Natural Sciences, Tampere University, 33820 Tampere, Finland

*Corresponding author: mikhail.vagin@liu.se, +46702753087

**Table of Contents**

1. Experimental section: page 3
2. **Figure** **1S**: page 7
3. **Figure** **2S**: page 8
4. **Supporting Note 1** (GIWAXS): page 9

- Figure 3S: page 9

1. **Figure 4S**: page 10
2. **Supporting Note 2** (Effect of secondary doping on the performance of organic electrochemical transistors (OECT)): page 11

- Figure 5S: page 12
- Figure 6S: page 13

1. **Figure 7S**: page 14
2. **Supporting Note 3** (Electrochemically active surface area (EASA) and ORR kinetics on PEDOT films by electrochemical impedance spectroscopy): page 15

- Figure 8S: page 18
- Figure 9S: page 20
- Figure 10S: page 21
- Table 1S: page 22
- Figure 11S: page 24

1. **Figure 12S**: page 25
2. **Supporting Note 4** (Koutecky-Levich study): page 26
3. **Supporting Note 5** (Processing of currents recorded on platinum ring detector electrode): page 27
4. **Figure 13S**: page 28
5. **Figure 14S**: page 29
6. **Figure 15S**: page 30-31
7. **Supporting Note 6** (Levich study): page 32

- Figure 16S: page 32

1. **Figure 17S**: page 33
2. **Figure 18S**: page 34
3. **Figure 19S**: page 35
4. **Figure 20S**: page 36

**Experimental section**

*Reagents and chemicals*

Dimethyl sulfoxide (DMSO, 99.9%), (3-glycidyloxypropyl)trimethoxysilane (GOPS), 97% ethylenedioxythiophene (EDOT), 5 wt.% Nafion perfluorinated ion exchange resin dispersion in water, hydrazine monohydrate and ammonium persulfate (APS) were purchased by Sigma-Aldrich. PEDOT:PSS (Clevios PH 1000) was purchased from Heraeus. High purity oxygen was supplied from Linde Gas AB. All reagents and chemicals were directly used without any purification processes. Deionized (DI) water was used in all experiments.

*Blend preparation*

The catalyst blends were prepared by adding various amounts of DMSO to the commercial aqueous dispersion Clevios PH1000. In order to obtain stability in water, 0.1 wt % GOPS was added to cross-link PEDOT:PSS. After sonication for 15 min, 5 μL of the catalyst blend was drop casted onto a glassy carbon disk surface and dried at 70 °C in oven, resulting in a mass loading of 0.26 mg cm^-2^.

*PEDOT:Nafion synthesis*

PEDOT:Nafion was prepared through oxidative polymerization of EDOT in a water dispersion of Nafion in the presence of the oxidizing agent APS, following a previously published protocol which was slightly modified [S. Carli et al., ACS Applied Materials & Interfaces, 2020, 12(26), 29807]. A mixture of EDOT (12 μL, 112 μmol), Nafion (0.5 mL) and water (1.5 mL) was vigorously stirred under nitrogen for 30 minutes. APS (25.98 mg, 113.84 μmol) was added in two portions and the resulting mixtures was stirred under N_2_ for 24 hours: the second portion (≈ 50% of the overall amount of APS) was added after 7 hours. The reaction mixture was diluted with water and the solid was purified by centrifugation method. The supernatant was separated, water was added, and the overall process was repeated 6-7 times to remove the excess of unreacted EDOT as well as inorganic impurities. The purification protocol was monitored by UV-Vis spectroscopy and continued until the UV spectra of the PEDOT:Nafion dispersion (Fig. 1SA) reached a steady state. Finally, after the last purification step, PEDOT:Nafion was dispersed in water (3 mL) and sonicated at room temperature for 20 minutes, to yield a solid content on the order of 1.44 wt. %.

*PEDOT:Nafion Raman characterization*

An Edinburgh FS920 spectrofluorimeter was used to collect Raman spectra of PEDOT:Nafion film. The instrument was equipped with a 189 mW CW 532 nm laser as the excitation source and a photomultiplier tube as the detector. The excitation bandwidth was 0.02 nm, while the emission slit was set at 0.1 nm. 200 scans sampled at a 0.1 nm step were averaged in order to achieve an acceptable S/N ratio. The sample was prepared by drop casting the dispersion of PEDOT:Nafion on a conductive glass substrate (FTO TEC-7, bought from NSG) and water was allowed to evaporate under room temperature. The Raman measurement of the reduced form of PEDOT:Nafion was collected after chemical reduction of the film by a hydrazine solution [N. Massonnet et al., Journal of Material Chemistry C, 2014, 2(7), 1278]. A diluted solution of hydrazine in water (32 wt. %) was dropped on top of the PEDOT:Nafion film on a hot plate (50°C) to ensure full coverage of the coating. After 30 minutes the reduced PEDOT:Nafion film was warmed to room temperature and Raman spectra were collected.

*X-ray photoelectron spectroscopy (XPS)*

XPS was performed on PEDOT:Nafion films using Scienta-200 hemispherical analyzer using monochromatized Al Kα source with photon energy of 1486.6 eV. The experimental conditions were such that the FWHM of the clean Au4f7/2 line was 0.65 eV. All photoelectron spectroscopy measurements were carried out with a base pressure lower than 1 × 10^-9^ mbar.

*Electrochemical measurements*

All electrochemical measurements were performed using a Bio-Logic SP-300 bi-potentiostat. A rotating ring disk electrode (RRDE, Pine Inc.) with control of rotation speed was used. The glassy carbon disk electrode (0.2475 cm^2^ in area) and platinum ring electrode (0.1866 cm^2^ in area) were used as two working electrodes. All the measurements were performed with a three-electrode cell. A platinum wire was used as the counter electrode. The absence of the platinum contamination [G. Jerkiewicz, ACS Catalysis, 2022, 12(4), 2661] was confirmed by the identity of voltammetry curves independently obtained using either carbon felt or platinum wire as counter electrodes (Fig. 2S). Hg/HgO (1M KOH) electrode was used as the reference electrode. All the measurements were performed at room temperature in 0.1M KOH (pH 13) or 0.1M HClO4 (pH 1.0).

All the measured potentials were calculated vs reversible hydrogen electrode (RHE) using the following equation: $E_{RHE}=E_{Hg/HgO}+0.0591\times pH+0.098$, where $E_{Hg/HgO}$ is the potential measured vs Hg/HgO (1M KOH). For 0.1M HClO_4_ the measured potentials were recalculated vs RHE using the following equation: $E_{RHE}=E_{Ag/AgCl}+0.0591\times pH+0.1976$, where $E_{Ag/AgCl}$ is the potential measured vs Ag/AgCl (3M KCl). The ORR measurements were conducted by linear sweep voltammetry from 1.35 V to -0.13 V (RHE) in oxygen- or argon-saturated electrolyte at a scan rate of 20 mV s^-1^ with electrode rotation rates from 0 to 2500 rpm. The potential on the platinum ring electrode was maintained at 1.2 V (RHE).

*Resistometry measurements*

*Ex-situ* electrical conductivity was measured on PEDOT films spin-coated (600 rpm) on the 1.5 × 1.5 cm glass substrates by a four-point probe method. The thickness (ca. 80 nm) of deposited films were estimated by optical profilometer. *In-situ* resistometry was carried out on a two-terminal gold interdigitated microelectrode array on glass (15 μm gap; MicruX Technologies, Spain) in argon-saturated 0.1M KOH. The array was modified by PEDOT films by drop-casting. Hg/HgO electrode and platinum mesh were used as the reference and counter electrodes. A bi-potentiostat was used to control two independent working electrodes, namely the two terminals of the interdigitated microelectrode array. The channel current was calculated by means of subtraction of current recorded on first working electrode with 0 mV bias (with respect to the second working electrode) from the current recorded on first working electrode with 50 mV bias. The apparent film resistance was calculated as a quotient of 50 mV bias to channel current.

*Measurements on gas-diffusion electrodes*

# 50 μL PEDOT:PSS blends were drop casted on a disk (2 cm diam.) of carbon paper (0.19 mm thickness, 78% porosity, AvCarb MGL190, Fuel Cell Store). The modified disk was dried at ambient conditions for at least 60 minutes and clamped between stainless-steel body with engraved gas flow-field and PTFE lid of the gas flow cell (Inset in Fig. 6A, GDE Cell, Greifswald, Germany). Both gas inlet and outlet were connected to flow meters to maintain a constant gas supply (0.2 L min^–1^). The catalytic surface of the modified disk was exposed (disk 3 mm diam.) to the electrolyte solution. 5 mL of 0.1M KOH electrolyte was added to the PTFE lid. A platinum mesh placed in an electrolyte-filled compartment separated by a porous glass frit and Hg/HgO electrode were used as counter and reference electrodes, respectively.

*H_2_O_2_ assaying*

Aliquots (volumes from 50 μL to 1 μL depending on H_2_O_2_ concentration) were collected from the gas flow cell for each hour of electrolysis. Then stirred freshly prepared solution of horseradish peroxidase (HRP, 0.75 ng ml^-1^) and 3,3′,5,5′-tetramethylbenzidine (TMB, 30 mg ml^-1^) in 0.1 M phosphate-citrate buffer solution (pH 6) was added to each aliquot up to the total volume of 300 μL. Then the absorbance of the mixture was measured at 653 nm by a UV-vis plate reader (BioTek Synergy H1 Hybrid Multi-Mode Reader). Finally, the concentration of H_2_O_2_ was determined from the calibration line with known H_2_O_2_ concentrations (0 μM, 10 μM, 20 μM, 30 μM, and 40 μM) by standard additions method.

**Figure 1S.** UV-vis absorbance spectra of PEDOT:Nafion dispersion (**A**) and Raman spectra of PEDOT:Nafion films (**B**; doped and dedoped as black and red curves, respectively).

**Figure 2S.** The absence of the effect of the platinum counter electrode. **A:** сyclic voltammograms obtained on glassy carbon rotating disk electrode modified by PEDOT:PSS (8% DMSO, 1600 rpm, curves obtained with carbon felt and platinum wire counter electrode are rad and blue curves, respectively); **B:** voltammetry on rotating disk ring electrode (1600 rpm): solid curves - background-subtracted currents obtained on glassy carbon rotating disk electrode modified by PEDOT:PSS (8% DMSO), dashed curves - voltammograms obtained on platinum ring electrode; scan rate 20 mV s^-1^, oxygen-saturated 0.1M KOH.

**Supporting Note 1**

**Grazing Incidence Wide Angle X-ray Scattering (GIWAXS)**

The experiments were performed on the Dutch-Belgian Beamline (DUBBLE CRG), station BM26B, at the European Synchrotron Radiation Facility (ESRF), Grenoble, France. The energy of the X-rays was 12.42 keV, and the sample-to-detector distance was set as 12.3 cm. The angle of incidence, α_i_, was set at 0.16°. The diffracted intensity was recorded by a Frelon CCD camera and was normalized by the incident photon flux and the acquisition time. Flat field, polarization, solid angle, and efficiency corrections were subsequently applied to the 2D GIWAXS images. The scattering vector q was defined with respect to the center of the incident beam and has a magnitude of *q = (4π/λ)sin(θ)*, where *2θ* is the scattering angle and *λ* is the wavelength of X-ray beam (0.998 Å). Wedge correction was applied to obtain the *q_z_* versus *q_r_* images, where *q_r_* and *q_z_* are the in-plane and near out-of-the-plane scattering vectors, respectively. Next, an azimuthal integration was performed on the 2D images to obtain the corresponding 1D scattering patterns. 1D scattering patterns are presented in after background subtraction.

**Figure 3S.** The evolution of the 1D GIWAXS patterns with increase in DMSO content in PEDOT:PSS blends. Scattering patterns are presented after applying normalization and background subtraction.


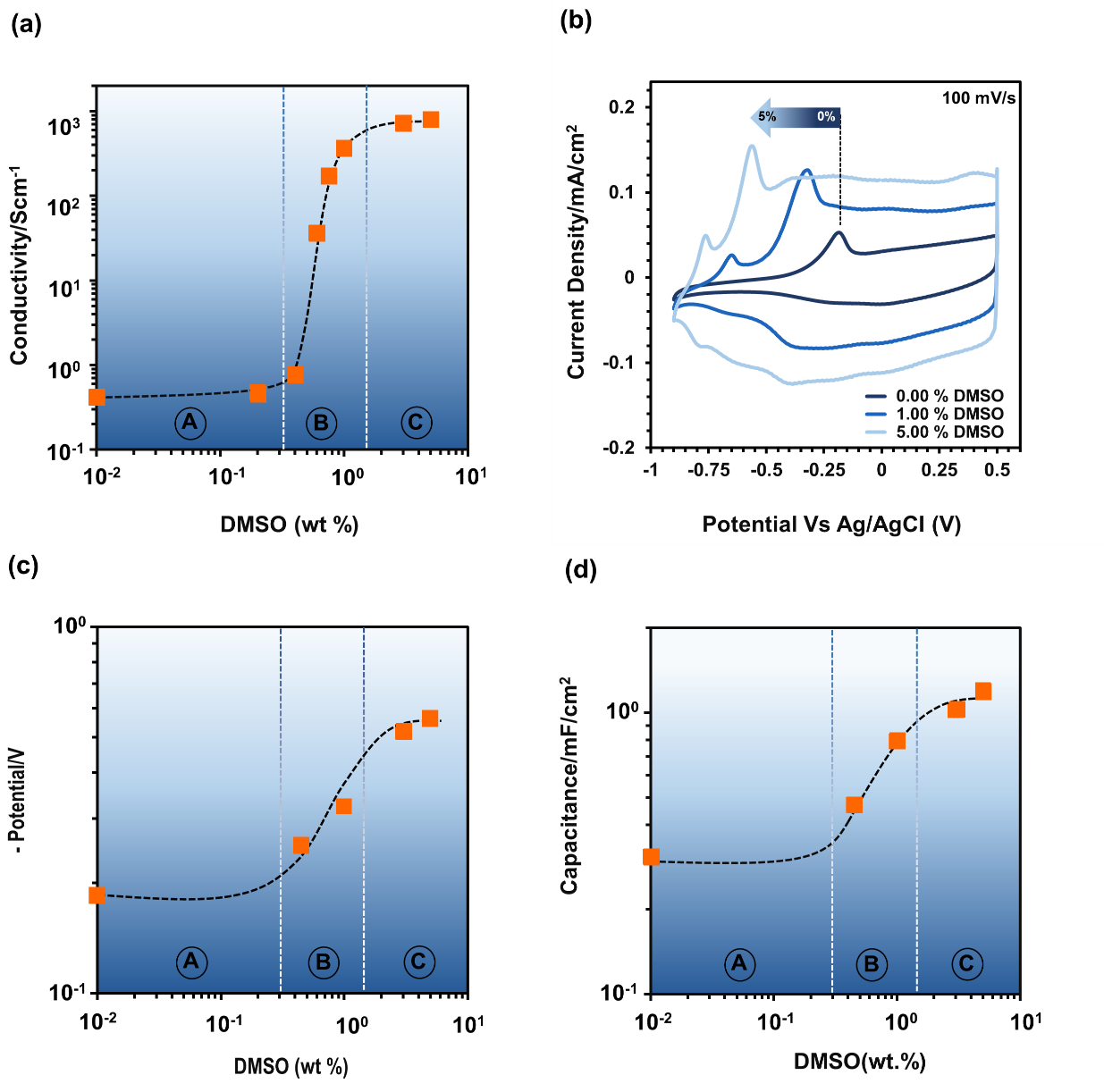


**Figure 4S.** The extension of conductivity window and increase of electrocapacitive currents upon secondary doping of PEDOT:PSS films (0.1 M Na_2_SO_4_, 20 mV s^-1^).

**Supporting Note 2**

**Effect of secondary doping on the performance of organic electrochemical transistors (OECT)**

To fabricate OECTs (Fig. 5S(a)), PEDOT:PSS blends with different DMSO content were spin coated on pre-patterned glass substrates with gold electrodes (50 nm thickness) sputtered on chromium (5 nm thickness). The channel dimensions were 25 μm long and 100 μm wide. On top of the PEDOT:PSS layer, a thin protective layer of PVDF-HFP was spin coated at 2000 rpm for 30 seconds using a solution (4mg ml^-1^) in ethyl ketone. The films were annealed at 100 ^o^C for 90 seconds. A layer of Shipley s1818 photoresist was spin coated at 2000 rpm for 60 seconds, which was followed by annealing at 120 ^o^C for 90 seconds. After exposure to UV light, the devices were dipped at a MF-319 developer solution to remove the exposed photoresist. The patterned devices were then etched with plasma reactive ion etching in oxygen/tetrafluoromethane for 60 seconds. The etched devices were washed with acetone to remove the PVDF-HFP layer and then a thick SU-8 2002 layer was deposited and patterned. The devices were then rinsed with iso-propanol to remove the unreacted SU-8. A PDMS ring of 50 μm thickness was then deposited on top of the device to form the well for the electrolyte solution. A Ag/AgCl electrode was used as a gate electrode for the OECT operation. The devices were evaluated with a Keithley 4200 semiconductor parameter analyzer.

The fabricated OECTs were evaluated in a neutral aqueous electrolyte 0.1 M Na_2_SO_4_. A system with a lower density (PEDOT:PSS with 0 vol% DMSO) can be easily dedoped at a lower gate voltage, severing the percolation path and the charge transport of the charge carriers. When the content of the secondary dopant increased, a higher gate voltage was needed to completely de-dope the channel to reach negligible drain currents, which is evident from the transfer curves obtained for the drain voltage of -0.1 V (Fig. 5S(b)). The turn-off voltage of OECTs is increasing in a sigmoidal form with increased DMSO content in the blend (Fig. 5S(c)), similarly to the electrical conductivity from 4-probe measurements on dried PEDOT:PSS films (Fig. 2A). The transconductance for PEDOT:PSS-based OECTs increases with increases in the secondary dopant content (Fig. 5S(d)).

We utilized UV-vis spectroelectrochemistry at different potentials applied on PEDOT:PSS films fabricated from blends with different secondary dopant contents (Fig. 6S). The selected potentials were applied for a long time in order to measure the film absorbance at the steady-state. The application of negative potentials resulted in the de-doping process represented by a decrease in the absorption typical for highly conductive state of the PEDOT:PSS. Importantly, no effects on doping level due to the secondary doping were observed on steady-state UV-vis absorption at the different applied potentials. This implies that if the external potential is applied for a sufficiently long time, the films remained unaffected by the secondary doping. Therefore, their degree of oxidation, or primary doping level, is not altered by the secondary doping.


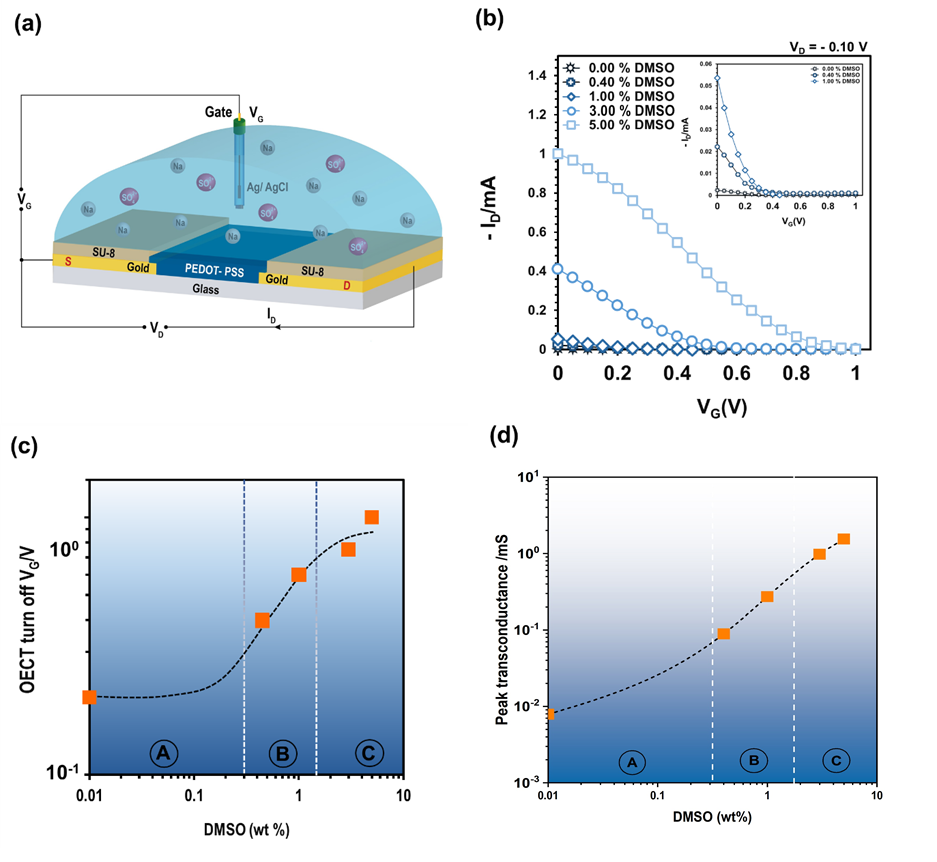


**Figure 5S.** **(a):** scheme of the OECT structure; **(b):** transfer curves recorded on PEDOT:PSS-based OECTs fabricated from the blend with different DMSO content (drain voltage -0.1 V; 0.1 M Na_2_SO_4_); **(c)** and **(d):** the dependencies of turn-off gate voltage and transconductance peak of PEDOT:PSS-based OECTs on the DMSO content.

**Figure 6S.** The absence of the secondary doping effects on the oxidation level of PEDOT:PSS at the steady-state. The UV-vis absorbance spectra were collected at different potentials (0.3 V (Ag/AgCl) and -0.9 V (Ag/AgCl), **upper** and **lower** figures, respectively) applied on ITO glass slides modified by PEDOT:PSS films fabricated from the blends of different DMSO content (10 minutes equilibration time, 0.1 M Na_2_SO_4_).
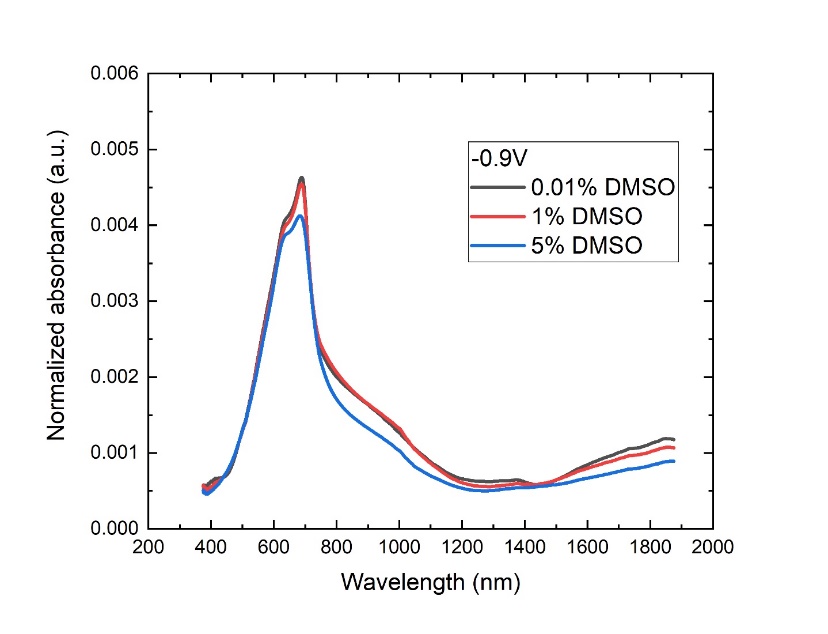

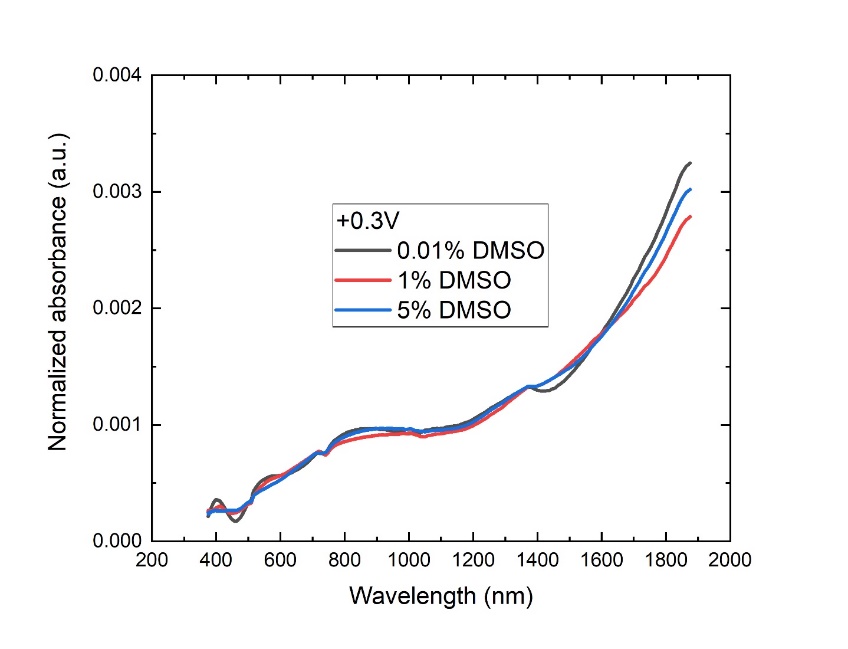

**Figure 7S.** The scaling-up of EASA with increased PEDOT:PSS film thickness. The dependence of the capacitive currents recorded at 0.8 V (RHE) on the loading of glassy carbon electrode with blend of PEDOT:PSS with 8 vol% DMSO.

**Supporting Note 3.**

**Electrochemically active surface area (EASA) and ORR kinetics on PEDOT films by electrochemical impedance spectroscopy**

To evaluate the EASA of PEDOT films, the impedance spectra of glassy carbon electrodes modified by PEDOT films were collected in capacitive region of potentials at 0.8 V (RHE). The real and imaginary parts of admittance were calculated, respectively, as:

$$Y^{'}=\frac{Z'}{\left( \left( Z^{'} \right)^{2}+\left( Z^{''} \right)^{2} \right)}$$

$$Y^{''}=\frac{Z''}{\left( \left( Z^{'} \right)^{2}+\left( Z^{''} \right)^{2} \right)}$$

where $Z'$ and $Z''$ are the real and imaginary parts of impedance (Ohms). The spectra were plotted in complex capacitance coordinates $Y'\left( 2\pi\upsilon\right)^{-1}$ vs $Y''\left( 2\pi\upsilon\right)^{-1}$ (Fig. 8SA), where $\upsilon$ is the frequency (Hz). The geometrical fit by a circle enabled the estimation of the values of total capacitance ($C_{total}$) of the PEDOT-modified electrodes. The increase in the DMSO content of the PEDOT:PSS blends as well as increase in the mass loading of glassy carbon electrodes by PEDOT:PSS blends resulted in the increase of the diameter of fitting circle confirming its assignment for the total capacitance of the film. In coherence with voltammetry (Fig. 7S), the increase of PEDOT:PSS mass loading resulted in a linear increase of total capacitance (Fig. 8SB) illustrating the linear scaling-up of EASA. The dependence of the total capacitance of PEDOT:PSS films on the secondary dopant (Fig. 8SC) content showed similar shape with the dependence of capacitive currents recorded on PEDOT:PSS films (Fig. 2C). The effect of the primary dopant is visible as more than 3 times increase of total film capacitance obtained for PEDOT:Nafion film compared with a PEDOT:PSS film of similar mass. The secondary doping of PEDOT:PSS led to the values of total capacitance similar to the capacitance obtained on PEDOT:Nafion.

To evaluate the ORR kinetics, the impedance spectra of glassy carbon electrodes modified by PEDOT films were collected at the region of low overpotentials of ORR, namely 0.6 V and 0.7 V (RHE). The equivalent circuits utilized for analysis (Insets in Fig. 9SB and 10SB) include solution resistance ($R_{S}$), two integrated RC elements and the total capacitance of the film ($C_{film}$). Similar equivalent circuits were developed for an electrode covered with a damaged (porous) coating [R.D. Armstrong et al., Corrosion Science, 1995, 37(10), 1615; M. O'Donoghue et al., Materials Performance NACE Int., 2003, 42(9), 36; D. Loveday et al., Journal of Coatings Technology and Research, 2005, 2(13), 22].

Importantly, we were not able to develop a unified equivalent circuit, which gave good fitting for all PEDOT films of both low and high conductivities. Specifically for the high conductivity films fabricated from the PEDOT:PSS blends with DMSO content of 1-8 % and PEDOT:Nafion, the equivalent circuit comprising the pure capacitances (Inset in Fig. 9SB) gave a value of the fitting quality parameter χ^2^ of ≤ 0.001 indicating a very good fit (Fig. 9SB-C). On the contrary, a good fit was obtained for films of low conductivity (PEDOT:PSS of 0 % and 0.3% DMSO, Fig. 10SB-C) only using an equivalent circuit comprising the constant phase elements ($CPE_{I}$ and $CPE_{II}$) instead of pure capacitances (Inset in Fig. 10SB), which illustrates an increase in the inhomogeneity of the porous interface of PEDOT:PSS films of low conductivity.

RC constants calculated for process I and II (Table 1S) are different, illustrating the difference in the rates of the corresponding processes. Specifically, for PEDOT films of high conductivity (PEDOT:Nafion and PEDOT:PSS formed from the blends with 1-8 vol%. DMSO), the RC constants for the process II are larger than for process I. This implies that the process I is faster than process II. The process II is faster than the process I on films of low conductivity (PEDOT:PSS formed from the blends with 0-0.3 vol%. DMSO). In contrast to $R_{I}$, the values of $R_{II}$ (Table 1S) fitted for impedance spectra obtained on films of high conductivity decrease with increasedof ORR driving force (from 0.7 V to 0.6 V (RHE)), which motivates the assignment of $R_{II}$ obtained on films of high conductivity to the charge transfer resistance originated from ORR kinetics. Then the process I on films of high conductivity would be the electronic transport within the conducting polymer, which is faster than the heterogeneous electron transfer in ORR. In analogy, $R_{I}$ associated with the slow process I on films of low conductivity (PEDOT:PSS formed from the blends with 0-0.3 vol%. DMSO) would be assigned to the charge transfer resistance originated from ORR kinetics.

The exchange current for ORR-to-H_2_O_2_, $i_{0}^{ORR-to-H2O2}$, the current in absence of the overpotential can be determined as:

$$i_{0}^{ORR-to-H2O2}=\frac{RT}{nFR_{CT}}$$

where $R$ is the gas constant (8.31 J mol^-1^ K^-1^), $T$ is the absolute temperature (298 ^o^K), $n$ is the number of transferred electrons (2 for ORR-to-H_2_O_2_), $F$ is Faraday constant (96485 s A mol^-1^) and $R_{CT}$ is the charge transfer resistance (Ohm; $R_{I}$ and $R_{II}$ for PEDOT films of low and high conductivity, respectively (Table 1S)). The normalization of ORR-to-H_2_O_2_ exchange currents on EASA represented by total capacitance ($C_{total}$, Fig. 8SC) enables the consideration of the effect of EASA on the ORR kinetics.

In analogy with voltammetry, the conductivity of PEDOT films modulated by primary and secondary doping has minor influence on the ORR kinetics if the effect of the EASA is considered (Fig. 11S). However, the inconsistency of the equivalent circuits utilized for fitting of impedance spectra obtained on PEDOT films of low and high conductivity challenges the unified study of the effect of film conductivity.

**Figure 8S.** Evaluation of EASA by electrochemical impedance spectroscopy. **A:** impedance spectra collected for PEDOT modified glassy carbon electrodes at 0.8 V (RHE) in 0.1M KOH in complex capacitance plot (amplitude 10 mV); **B:** the dependence of the total film capacitance on the mass loading of the glassy carbon electrode; **C:** the effect of secondary doping on the total capacitance of the PEDOT:PSS films (red open circle – PEDOT:Nafion).

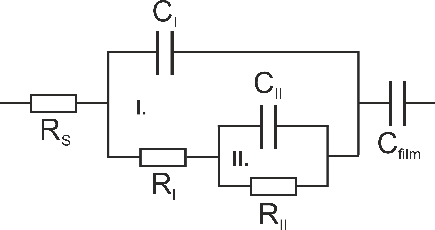

**Figure 9S.** Impedance spectra of high conductivity PEDOT films acquired in 0.1M KOH (amplitude 10 mV) at 0.7 V (RHE) in Nyquist (**A**) and Bode (**B** and **C**) coordinates; solid curves – spectra fitted using the equivalent circuit in Inset of **B**.

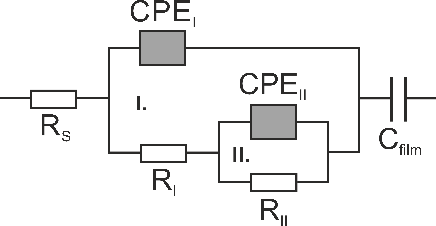

**Figure 10S.** Impedance spectra of low conductivity PEDOT films acquired in 0.1M KOH (amplitude 10 mV) at 0.7 V (RHE) in Nyquist (**A**) and Bode (**B** and **C**) coordinates; solid curves – spectra fitted using the equivalent circuit in Inset of **B**.

**Table 1S**. Results of fitting of experimental impedance spectra.

| Film | DMSO content | Potential (RHE) | $R_{s}$ | $P_{I}$  ×10^-4^ | $\phi_{I}$ | $C_{I}$ | $R_{I}$ | $R_{I}C_{I}$^***^ | $P_{II}$  ×10^-4^ | $\phi_{II}$ | $C_{II}$ | $R_{II}$ | $R_{II}C_{II}$^****^ | $C_{film}$ |
| --- | --- | --- | --- | --- | --- | --- | --- | --- | --- | --- | --- | --- | --- | --- |
|  | vol% | V | Ω |  |  | ×10^-6^ F | Ω | s |  |  | ×10^-3^ F | Ω | s | ×10^-3^ F |
| PEDOT:PSS | 0 | 0.6 | 37.1 | 12.8 | 0.5 | 60.0^*^ | 70.5 | 4230 | 9.0 | 0.85 | 0.49^**^ | 2855 | 1399 | 2.4 |
|  |  | 0.7 | 38.8 | 8.7 | 0.53 | 44.3^*^ | 71.3 | 3159 | 9.8 | 0.86 | 0.56^**^ | 3675 | 2058 | 2.3 |
|  | 0.3 | 0.6 | 37.7 | 44.3 | 0.38 | 238.5^*^ | 159 | 37921 |  |  | 0.24 |  |  | 2.6 |
|  |  | 0.7 | 40.1 | 12.2 | 0.51 | 68.3^*^ | 64.3 | 4391 | 9.6 | 0.84 | 0.52^**^ | 3241 | 1685 | 3.5 |
|  | 1 | 0.6 | 32.5 |  |  | 0.23 | 7.1 | 1.6 |  |  | 3.5 | 13.7 | 48.0 | 2.4 |
|  |  | 0.7 | 36.3 |  |  | 0.28 | 6.8 | 1.9 |  |  | 3.1 | 13.4 | 41.5 | 2.3 |
|  | 4 | 0.6 | 43.5 |  |  | 0.14 | 8.5 | 1.2 |  |  | 3.6 | 8.9 | 32.0 | 2.7 |
|  |  | 0.7 | 35.5 |  |  | 0.11 | 9.3 | 1.0 |  |  | 4.7 | 9.8 | 46.1 | 2.8 |
|  | 8 | 0.6 | 32.5 |  |  | 0.16 | 8.1 | 1.3 |  |  | 4.2 | 6.5 | 27.3 | 2.6 |
|  |  | 0.7 | 32.2 |  |  | 0.15 | 8.0 | 1.2 |  |  | 5.3 | 7.4 | 39.2 | 2.8 |
| PEDOT:Nafion | 0 | 0.6 | 34.0 |  |  | 0.14 | 8.3 | 1.2 |  |  | 3.8 | 7.8 | 29.6 | 3.2 |
|  |  | 0.7 | 34.6 |  |  | 0.14 | 8.4 | 1.2 |  |  | 3.9 | 8.4 | 32.8 | 3.3 |

^*^Defined as:$C_{I}=\left( {P_{I}\times\left( R_{S} \right)}^{\left( 1-\phi_{I} \right)} \right)^{1/\phi_{I}}$

where $R_{S}$ is the solution resistance, P is a fitting parameter of the CPE and $\phi$ is the fitted exponent factor, which varies from 0 to 1. When $\phi$ is tending to 0 the CPE behaves as a pure resistor, and when $\phi$ is tending to 1 the CPE represents a pure capacitor.

^**^Defined as:$C_{\mathrm{II}}=\left( {P_{\mathrm{II}}\times\left( R_{S} \right)}^{\left( 1-\phi_{\mathrm{II}} \right)} \right)^{1/\phi_{\mathrm{II}}}$

^***^Defined as $R_{I}C_{I}=R_{I}\times C_{I}$

^****^Defined as $R_{II}C_{II}=R_{II}\times C_{II}$

**Figure 11S.** The minor effect of PEDOT film conductivity on the ORR kinetics at 0.7 V (RHE). The dependence of EASA-normalized exchange current of ORR on the DMSO content in PEDOT:PSS blend. Red open symbol - EASA-normalized exchange current of ORR for PEDOT:Nafion.

**Figure 12S.** The absence of ORR activity on PEDOT:PSS in acidic electrolyte. The voltammograms recorded on RDE (1600 rpm) modified by PEDOT:PSS (8 vol% DMSO) in oxygen-saturated 0.1 M HClO_4_ or 0.1 M KOH.

**Supporting Note 4.**

**Koutecky-Levich study**

To estimate the kinetic currents, the family of voltammograms recorded on the rotating disk at different rotation speeds (Fig. 12S, left column (a, c, e, g, i, k, m and o)) were processed after subtraction of background voltammograms (i.e. recoded in nitrogen-saturated electrolyte) using the Koutecky-Levich equation:

$\frac{1}{I_{E}}=\frac{1}{I_{kinetic}}+\frac{1}{I_{L}}=\frac{1}{I_{K}}+\frac{1}{B\omega^{1/2}}$ (1S)

where $I_{E}$ is the total current recorded on rotating disk at the certain applied potential *E*, $I_{kinetic}$ is the kinetic current while $I_{L}$ is the current contribution due to diffusion limitation; *ω* is the angular rotation rate of the electrode (rad s^-1^) and *B* is a constant. The plots of the reciprocal total currents $\left( \frac{1}{I_{E}} \right)$ against the reciprocal root of the angular rotation rate $\left( \omega^{-1/2} \right)$ (KL plot, Fig. 12S, right column (b, d, f, h, j, l, n and p)) gives a straight line with a finite Y-axis intercept equal to the reciprocal kinetic current $\left( \frac{1}{I_{kinetic}} \right)$ at the certain potential *E* and the slope equal to $\left( \frac{1}{B} \right)$. The constant *B* in eq. (1S) is defined by the equation:

$B=0.201nFD_{0}^{2/3}v^{-1/6}C_{0}$ (2S)

where *n* is the number of transferred electrons per oxygen molecule, *F* is Faradic constant (96485 C mol^-1^), *A* is a geometrical surface area of the rotating disk electrode (0.24706 cm^-2^), *D*_0_ is the diffusion coefficient of oxygen (1.9 × 10^-5^ cm s^-1^ in 0.1M KOH), ν is the kinematic viscosity of the electrolyte (1 × 10^-2^ cm^2^ s^-1^), and *C_O_* is the bulk concentration of oxygen (1.2 × 10^-6^ mol cm^-3^).

**Supporting Note 5.**

**Processing of currents recorded on platinum ring detector electrode**

The currents recorded on platinum ring disk detector under the applied potential of H_2_O_2_ oxidation were processed using the equation:

$n =\frac{{4I}_{disk}}{I_{ring}/N + I_{disk}}$ (3S)

where $I_{ring}$ and $I_{disk}$ are the ring and the disk currents (A), respectively. The *in-situ* yield of H_2_O_2_ was calculated by the equation:

$H_{2}O_{2}\left( \% \right)=\frac{2I_{ring}}{NI_{disk}+I_{ring}}\times100\%$ (4S)

where $N$ is the collection efficiency estimated for standard single electron redox couple (ferricyanide, $N$ = 0.33).

**Figure 13S.** The comparable disk and ring currents (lower and upper parts, respectively) recorded on blank and PEDOT:PSS-modified (15 vol%) glassy carbon (oxygen-saturated 0.1 M KOH, 1600 rpm).

**Figure 14S.** The dependencies of mass-normalized kinetic currents of ORR on PEDOT:PSS films fabricated from blends of different DMSO content on applied potential (right column) and on film thickness (left column); oxygen-saturated 0.1M KOH.

f

h

a

b

d

c

e

g

j

i

l

k

m

n

p

o

**Figure 15S.** Linear sweep voltammograms and corresponding KL plots (left (a, c, e, g, i, k, m and o) and right columns (b, d, f, h, j, l, n and p), respectively) obtained for on PEDOT:PSS-modified glassy carbon disk electrode at different rotation rates with varying DMSO in PEDOT:PSS blends.

**Supporting Note 6.**

**Levich study**

The launching of rotation of PEDOT:PSS-modified disk electrode led to the appearance of the typical s-shaped curves (Fig. 12S, right column) featured with a region of potential-independent currents, so-called limiting currents below 0.1 V (RHE). The dependence of the limiting current on the angular frequency of rotation is described by Levich equation:

$I_{lim}=0.62nFAD^{2/3}\upsilon^{1/6}\omega^{1/2}C$ (5S)

****where $I_{lim}$ is the limiting current of ORR (A), $n$ is the number of transferred electrons ($n=2$ for ORR-to-H_2_O_2_), $F$ is the Faraday constant (96485 C mol^-1^), $A$ is the geometrical area of the rotating disk electrode (cm^2^), $D$ is the diffusion coefficient of oxygen (cm^2^ s^-1^), $\upsilon$ is the kinematic viscosity of solvent (0.0009 cm^2^ s^-1^ for water at 25 ^o^C), $\omega$ is the angular frequency of rotation (rad s^-1^, which is calculated as $\omega=\frac{2\pi q}{60}$, where $q$ is the rotation speed of rotating disk electrode (rpm)) and $C$ is the oxygen concentration in the bulk (1.2×10^-6^ mol cm^-3^ for 0.1 mM KOH). The diffusion coefficient of oxygen can be estimated for each film of PEDOT:PSS with different DMSO contents as the slope of linear dependence of the limiting current $I_{lim}$ vs $\omega^{1/2}$ (Levich plot, Fig. 16S).

**Figure 16S.** Levich study for ORR on PEDOT:PSS fabricated from blends with different DMSO content. The dependence of the limiting current (at 0.1 V vs RHE) on the square root of the angular frequency (Levich plot).

**Figure 17S.** The ORR activity on pristine carbon paper. The voltammograms recorded on the cell with gas diffusion electrode (Inset in Fig. 6A) assembled from blank unmodified carbon and fed by pure nitrogen, air and pure oxygen (0.1 M KOH).

Carbon Paper (CP)

**Figure 18S.** The diffusion limitation of ORR on pristine carbon paper. The time dependencies of the potentials on pristine carbon paper and carbon paper modified with PEDOT:PSS blended with DMSO (8 vol%). The spikes are due to the solution sampling for H_2_O_2_ quantification.

**Figure 19S.** The dependence of H_2_O_2_ production efficiency on air fed GDE on the current density.

**Figure 20S.** The time dependencies of H_2_O_2_ production efficiency and electric energy loss on air-fed GDE under constant current electrolysis at 12.5 mA cm^-2^.
